# Cognitive function based on theta-gamma coupling vs. clinical diagnosis in older adults with mild cognitive impairment with or without major depressive disorder

**DOI:** 10.1038/s41398-024-02856-5

**Published:** 2024-03-19

**Authors:** Heather Brooks, Wei Wang, Reza Zomorrodi, Daniel M. Blumberger, Christopher R. Bowie, Zafiris J. Daskalakis, Corinne E. Fischer, Alastair J. Flint, Nathan Herrmann, Sanjeev Kumar, Krista L. Lanctôt, Linda Mah, Benoit H. Mulsant, Bruce G. Pollock, Aristotle N. Voineskos, Tarek K. Rajji, the PACt-MD Study Group, Benoit H. Mulsant, Tarek K. Rajji, Nathan Herrmann, Bruce G. Pollock, Daniel M. Blumberger, Christopher R. Bowie, Meryl A. Butters, Corinne E. Fischer, Alastair J. Flint, Angela Golas, Ariel Graff, James L. Kennedy, Sanjeev Kumar, Krista L. Lanctôt, Lillian Lourenco, Linda Mah, Shima Ovaysikia, Mark Rapoport, Kevin E. Thorpe, Nicolaas P. L. G. Verhoeff, Aristotle Voineskos

**Affiliations:** 1 Campbell Family Mental Health Research Institute, Centre for Addiction and Mental Health, Toronto, ON Canada; 2 Adult Neurodevelopment and Geriatric Psychiatry Division, Centre for Addiction and Mental Health, Toronto, ON Canada; 3 Temerty Centre for Therapeutic Brain Intervention, Centre for Addiction and Mental Health, Toronto, ON Canada; 4 Department of Psychiatry, Temerty Faculty of Medicine, University of Toronto, Toronto, ON Canada; 5 Department of Psychology, Queen’s University, Kingston, ON Canada; 6 Keenan Research Centre for Biomedical Science, St. Michael’s Hospital, Toronto, ON Canada; 7 Centre for Mental Health, University Health Network, Toronto, ON Canada; 8 Sunnybrook Health Sciences Centre, Toronto, ON Canada; 9 Toronto Dementia Research Alliance, University of Toronto, Toronto, ON Canada; 10 Rotman Research Institute, Baycrest Health Sciences Centre, Toronto, ON Canada; 11 Applied Health Research Centre, Li Ka Shing Knowledge Institute, St. Michael’s Hospital, Toronto, ON Canada; 12 Molecular Science Department, Centre for Addiction and Mental Health, Toronto, ON Canada; 13 Evaluative Clinical Sciences, Hurvitz Brain Sciences Research Program, Sunnybrook Research Institute, Toronto, ON Canada; 14 Department of Psychiatry, University of Pittsburgh School of Medicine, Pittsburgh, PA USA; 15 Brain Health Centre, Baycrest Health Sciences, Toronto, ON Canada

**Keywords:** Physiology, Molecular neuroscience

## Abstract

Whether individuals with mild cognitive impairment (MCI) and a history of major depressive disorder (MDD) are at a higher risk for cognitive decline than those with MCI alone is still not clear. Previous work suggests that a reduction in prefrontal cortical theta phase-gamma amplitude coupling (TGC) is an early marker of cognitive impairment. This study aimed to determine whether using a TGC cutoff is better at separating individuals with MCI or MCI with remitted MDD (MCI+rMDD) on cognitive performance than their clinical diagnosis. Our hypothesis was that global cognition would differ more between TGC-based groups than diagnostic groups. We analyzed data from 128 MCI (mean age: 71.8, SD: 7.3) and 85 MCI+rMDD (mean age: 70.9, SD: 4.7) participants. Participants completed a comprehensive neuropsychological battery; TGC was measured during the N-back task. An optimal TGC cutoff was determined during the performance of the 2-back. This TGC cutoff was used to classify participants into low vs. high-TGC groups. We then compared Cohen’s *d* of the difference in global cognition between the high and low TGC groups to Cohen’s *d* between the MCI and MCI+rMDD groups. We used bootstrapping to determine 95% confidence intervals for Cohen’s *d* values using the whole sample. As hypothesized, Cohen’s *d* for the difference in global cognition between the TGC groups was larger (0.64 [0.32, 0.88]) than between the diagnostic groups (0.10 [0.004, 0.37]) with a difference between these two Cohen’s *d’*s of 0.54 [0.10, 0.80]. Our findings suggest that TGC is a useful marker to identify individuals at high risk for cognitive decline, beyond clinical diagnosis. This could be due to TGC being a sensitive marker of prefrontal cortical dysfunction that would lead to an accelerated cognitive decline.

## Introduction

Mild cognitive impairment (MCI) is typically a transitional stage towards dementia [1, 2]. It is not uncommon for individuals with MCI to have comorbid major depressive disorder (MDD; [3]). Whether a comorbid MDD with MCI increases the risk of progression to dementia in individuals is not clear, especially among those with remitted MDD (rMDD). In one study, individuals with MCI with active depressive symptoms had an increased risk of progression over an average of 2.6 years of follow-up, but past history of depression did not have an impact on the risk of progression [4]. Consistently, another study [5] found no difference between individuals with MCI and individuals with MCI+rMDD on general cognition assessed using the Mini-Mental State Examination (MMSE) [6]. In contrast, a third study found that individuals with MCI and active depression were more cognitively impaired than those without active depression [7]. However, they did not improve on verbal fluency a 1-year after their depression improved, although they improved on a calculation task. Finally, a study comparing cognitive performance across several cognitive domains found that individuals with amnestic MCI and amnestic MCI+rMDD had similar impairment across some tests of executive function, processing speed, and memory compared to a group of control participants. However, those MCI+rMDD showed significant deficits in a language measure, a visuospatial measure, and an executive function measure compared to those with MCI alone [8].

Thus, ascertaining whether an individual with MCI has a comorbid active or rMDD does not necessarily help in determining whether this individual is at a higher risk for cognitive decline or dementia. Consequently, an alternative approach to classifying individuals with MCI, with or without MDD, into those at higher or lower risk for cognitive decline or dementia is needed.

Theta-gamma coupling (TGC) is a neurophysiologic mechanism associated with an ordering of information in various cognitive functions [9–12]. We have shown that prefrontal cortex TGC predicts performance on various cognitive tasks that require ordering across individuals with MCI, rMDD, and MCI+rMDD, independent of diagnoses [9, 13]. We have also shown that prefrontal cortex TGC during a working memory task is impaired in individuals with MCI even when working memory performance was preserved [13]. This finding suggests that TGC is more sensitive to prefrontal cortical dysfunction than behavioral performance. Taken together, prefrontal cortex TGC could be a neurophysiologic marker of prefrontal cortical functioning that is better at identifying individuals with MCI, with or without rMDD, that are at a high risk for cognitive decline or dementia than clinical diagnosis.

As a first step towards addressing the above question, we hypothesized in this study that—using cross-sectional data—global cognitive function would differ more between groups defined by a TGC cutoff than between groups defined by the clinical diagnoses of MCI vs. MCI+rMDD. We also explored whether the groups based on the TGC cutoff would separate better on individual cognitive domains (verbal memory, visuospatial memory, processing speed, language, working memory, and executive function) than the groups based on clinical diagnosis.

## Materials and methods

### Participants

Participants were recruited as part of the PACt-MD study (Prevention of Alzheimer’s Dementia with Cognitive Remediation plus Transcranial Direct Current Stimulation in Mild Cognitive Impairment and Depression; NCT02386670) across five academic hospitals in Toronto, Canada. A total of 211 participants with MCI or MCI+rMDD and 78 non-psychiatric ‘healthy’ controls were included in this analysis. Full details on the sample have been published elsewhere [9, 14]. Briefly, participants with MCI met the following inclusion criteria: (1) age 60 years and older, (2) a diagnosis of MCI based on the Diagnostic and Statistical Manual of Mental Disorders 5 (DSM-5) criteria, and (3) never met DSM-5 criteria for a major depressive episode (MDE). Those with MCI+rMDD met the following criteria: (1) aged 65 years or older, (2) a diagnosis of both MCI and rMDD based on the DSM-5 criteria with an MDE that occurred after the age of 18 with: (a) an offset of 2 months to 5 years before the screening visit, or (b) an offset of 5 years or longer before the screening visit, with at least one episode requiring medical attention (e.g., saw a psychiatrist or primary care physician; received antidepressants or was hospitalized), and (3) not having been treated with electroconvulsive therapy during the past 6 months. None of the participants met the following exclusion criteria: (1) having ever met DSM-5 criteria for schizophrenia, bipolar disorder, or obsessive-compulsive disorder (OCD), (2) having met DSM-5 criteria for alcohol or other substance use disorder in the last 12 months, (3) presence of unstable physical illnesses or significant neurological conditions (e.g., stroke, seizures), (4) having taken a cognitive enhancer (e.g., acetylcholinesterase inhibitor) within the past 6 weeks, and (5) having a Montgomery-Äsberg Depression Rating Scale (MADRS) [15] score of 11 or more.

A group of non-psychiatric control participants was recruited using the following eligibility criteria: (1) aged 60 years and older, (2) no lifetime history of any DSM-5 diagnoses, with the exception of specific phobias, (3) no significant neurological conditions (e.g., stroke, seizures, etc.) or unstable physical conditions (e.g., uncontrolled hypertension), (4) not taking any psychotropic medications, except for zopiclone up to 15 mg/day, trazodone up to 150 mg/day, a benzodiazepine up to 3 mg/day lorazepam-equivalents, or gabapentin or pregabalin if prescribed for pain. All participants provided written informed consent using a form approved by the local Research Ethics Board prior to completing any study-related procedures.

### Assessments

#### Clinical and cognitive assessments

All participants were assessed using the Structured Clinical Interview for the Diagnostic and Statistical Manual 5 (SCID-5) [16], the MADRS [15], the Montreal Cognitive Assessment (MoCA) [17], and MMSE [6]. They also completed a comprehensive neuropsychological battery (Table 1) that assessed verbal memory using the California Verbal Memory Test-II (CVLT-II; [18]); visuospatial memory using the Brief Visuospatial Memory Test—Revised (BVMT-R; [19]); processing speed using the Digit Symbol Coding (DSC; [20]) test and the Trail Making Test (TMT) Part A [21]; working memory using the Paced Auditory Serial Addition Test (PASAT; [22]) and the Continuous Performance Test—Identical Pairs (CPT-IP; [23]); language using the Boston Naming Test (BNT; [24]), semantic fluency (animals), and letter fluency (F, A, and S); and executive function using the TMT Parts A and B [21], the Stroop Color-Word Test [25], and the Clock Drawing Test (CDT; [26]). The scores for each test for each participant were converted into z scores using the mean and standard deviation from the non-psychiatric control group. As previously described in detail [14], cognitive domain composite scores were generated by averaging the z scores for each individual test for each participant, and a global cognition composite score was generated by averaging the six cognitive domain scores (see Table 1): verbal memory, visuospatial memory, processing speed, language, working memory, executive function.

**Table 1:** List of neuropsychological tests and their corresponding cognitive domains.

Cognitive domain	Neuropsychological tests
Verbal memory	CVLT-II Total Recall (trials 1–5) CVLT-II *d’* CVLT-II % Retained
Visuospatial memory	BVMT-R Total Recall (trials 1–3) BVMT-R % Retained
Processing speed	Digit Symbol Coding Correct Responses TMT A Time per Connection
Working memory	PASAT Correct Responses on 2.4 and 1.6 s versions CPT-IP average *d’* on the 2-, 3-, and 4-digit tasks
Language	BNT Total Correct Responses Semantic Fluency (Animals) Total Words Letter Fluency (FAS) Total Words
Executive function	TMT B/A Ratio Stroop Color Word Correct Responses Clock Drawing Test Total Score

*CVLT-II* California verbal learning test-II, *BVMT-R* Ben visuospatial memory test-revised, *TMT* trail making test, *PASAT* paced auditory serial addition test, *CPT-IP* continuous performance test identical pairs, *BNT* Boston naming test.

#### N-back task

The N-back task is a continuous working memory task for which participants must determine if the stimulus presented on the screen is the same as, or different from, the stimulus presented N trials back. Our experimental set-up has been published in full elsewhere [13, 27]. In our task, *N* varies from 0 to 3, allowing us to index working memory at varying cognitive loads. In this analysis, the primary behavioral outcome was *d’*, which is calculated as: *d’* = z(Hits) – z(False Alarms). As in our other publications using the same group of participants [9, 13], we chose the 2-back as the primary condition, as it better indexes working memory than the 0- and 1-back [28], but individuals with cognitive impairment can perform it, and still generates meaningful performance compared to the 3-back [29].

### EEG recording and processing

During the N-back task, EEG is recorded using a 64-channel Synamps 2 EEG system and the 10–20 montage system, where electrodes were referenced to an electrode posterior to Cz. EEG signals were recorded using DC and a low pass filter of 100 Hz at 1-kHz sampling rate. Data cleaning and processing occurred offline using MATLAB (The MathWorks, Inc.) and EEGLab toolbox. An independent component analysis (ICA; EEGLAB toolbox; Infomax algorithm) was run to remove noise from the data, including eye blinks and muscle artifacts. Our EEG set-up is identical to the setups previously described [9, 13].

### Theta-gamma coupling

The process for calculating the modulation index (MI)—the measure of TGC—has been described elsewhere [9, 10, 13, 27]. The modulation index was calculated at each electrode, and then averaged across the frontal electrodes (F7/8, F5/6, F3/4, F1/2, and Fz). We then created a weighted MI value across all four trial results on the N-back task (i.e., target correct, target non-correct, non-target correct, and non-target non-correct). We created this weighted value based on the number of epochs of each trial result during the 2-back. For each trial result (i.e., target correct, target non-correct, non-target correct, and non-target non-correct), we multiplied the percent of epochs of that trial result over the entire task by the MI value for that trial result. Then, we took the average of these four values to generate one MI value that is weighted by trial result.

### Statistical analyses

All data were analyzed using the Statistical Program for Social Sciences (SPSS) version 25.0 [30] and RStudio [31]. Data were checked for normal distribution, and outliers ±3 SDs from the mean were removed from the analysis.

We compared the demographic, clinical, neuropsychological, and neurophysiologic measures in the two diagnostic groups (i.e., MCI and MCI+rMDD) with independent samples *t* tests or chi-square tests.

#### Determining the TGC Cutoff

To find the optimal TGC cutoff to use for the cognitive composite scores analyses, we first categorized the whole sample into “impaired” and “not impaired” based on their 2-back performance. To categorize them, and as cognitive performance is known to decline with age, we first generated age-expected *d’* scores with the regression equation from a linear regression model in the non-psychiatric control group with age as the independent variable and 2-back *d’* as the dependent variable. To generate age-corrected z scores, we subtracted the age-expected *d’* scores from the participants true *d’* score and divided it by the standard deviation of the residuals from the control regression equation. We used −1 as our cutoff, such that anyone who had an age-corrected *d*’ *z* score ≤−1 was classified as a “2-back impaired” and anyone with an age-corrected *z* score of >−1 was classified as a “2-back not impaired”. One standard deviation cutoff was chosen as it was also the cutoff used to ascertain impairment on the neuropsychological tests in the parent study and historically to indicate at least mild impairment in neuropsychological practice [32].

We then used the Youden Index (*J*), which combines sensitivity and specificity, as the objective function to determine an optimal TGC cutoff value. Here the Youden Index *J* is defined as a function of the cutoff value *c*:

1 $$J(c)={Sensitivity}\left(c\right)+{Specificity}\left(c\right)$$

The cutoff that achieves the maximum of *J*(*c*), is referred to as the optimal cutoff. It is the cutoff that optimizes the differentiating ability when equal weight is given to the sensitivity and specificity [33]. This step of determining the optimal TGC cutoff value that best separates participants into impaired or not impaired 2-back performers was done in the whole sample of MCI and MCI+rMDD (*n* = 211).

#### Using the TGC cutoff to determine cognitive performance

Using this optimal TGC cutoff determined above, we then evaluated how well the cutoff separated participants on the global cognition composite (primary analysis) and the individual cognitive domains (exploratory analyses). We calculated Cohen’s *d* values for the differences in the global cognition composite between the TGC groups (i.e., high-TGC group vs. low-TGC group) and between the diagnostic groups (i.e., MCI vs. MCI+rMDD). Then, we calculated Cohen’s *d* values for differences in the cognitive domain scores both between the TGC groups and the diagnostic groups. The Cohen’s *d* values for the difference between TGC and diagnosis were our primary outcome measure.

Lastly, we used bootstrapping (*n* = 5000), drawing a sample of 211 samples for each iteration, to generate 95% confidence intervals around our estimates. For each iteration, the TGC cutoff that best separated that sample of 211 into “impaired” vs. “not impaired” on the 2-back using the Youden Index was generated, and subsequently tested on the cognitive composite scores. We used these data to generate 95% CIs around the TGC cutoff, sensitivity, specificity, the Youden Index, and Cohen’s *d* values to evaluate the variability in these measures. Of note, when a pair of CIs presents an overlap, it does not necessarily indicate that the difference between the two Cohen’s *d’*s are not significantly different since the two Cohen’s *d’*s are based on the same sample of observations (thus positively correlated). To determine whether the difference between the Cohen’s *d*’s (the one based on the TGC cutoff and the other based on the clinical diagnosis) is significant, we examined the 95% CIs of the difference in these two Cohen’s *d*’s and whether these 95% CIs overlap with 0 or not.

#### Cross-validation analysis

It is important to note that TGC cutoff was generated using 2-back performance, and 2-back performance was not included as a test to generate any of the cognitive domain scores (please see Table 1 for tests used to generate cognitive composite scores). Still, because we use a cognitive test (2-back) to determine the TGC cutoff and we use the same sample for this determination as the one we use to test the ability of the TGC cutoff to separate high and low TGC groups on various cognitive function, we conducted a cross-validation analysis by splitting the sample into training and validation sub-samples to generate and test the TGC cutoff in independent samples.

We first created a bootstrapped sample with *n* = 211, drawing from our original sample, with replacement. The bootstrapped sample was then randomly split in half, and one half was designated as the training sample, and the other as the validation sample. The training sample was used to generate the TGC cutoff as described above for the full sample. The TGC cutoff was then tested in the validation sample, as also described above for the full sample. This process was repeated a total of 5000 times, each time with a different bootstrapped sample, and a different random assignment into training and validation samples. Compared to the conventional cross-validation method that repeatedly splits the same sample, the bootstrapping-based method generated independent performance measures that can be used to estimate the variability of the TGC cutoff performance. Codes used for analyses can be accessed by request.

## Results

Demographic, clinical, neurophysiologic, and neuropsychological variables are presented in Table 2.

**Table 2:** Demographic, clinical, neurophysiologic, and neuropsychological measures in the diagnostic groups.

	Diagnosis		
	MCI (*n* = 128)	MCI + rMDD (*n* = 83)	*t or χ*^*2*^ (df), *p*
Age	71.96(7.23)	70.83(4.74)	1.37 (209), 0.17
Highest level of education			0.72 (4), 0.95
Less than high school	6	4	
High school graduate	13	9	
Partial University	10	9	
University degree	66	42	
Graduate degree	33	19	
Gender (M:F)	50:78	31:52	0.06 (1), 0.80
MMSE	27.90 (1.62)	28.06 (1.56)	−0.72 (207), 0.47
MoCA	23.80 (2.43)	24.70 (2.61)	−2.56 (208), 0.01*
2-back TGC	0.0018 (0.0013)	0.0017 (0.0010)	0.56 (209), 0.56
Age-corrected 2-back *d’* *z* score	−1.12 (1.23)	−1.40 (1.38)	1.58 (209), 0.12
Global cognition composite	−0.85 (0.78)	−0.78 (0.72)	−0.63 (209), 0.53
Verbal memory composite	−1.07 (1.27)	−0.81 (1.08)	−1.54 (205), 0.13
Visuospatial memory composite	−0.84 (1.33)	−0.65 (1.08)	−1.14 (205), 0.26
Processing speed composite	−0.80 (1.15)	−1.05 (1.22)	1.50 (209), 0.13
Working memory composite	−0.80 (0.91)	−0.80 (0.97)	0.10 (209), 0.99
Language composite	−0.91 (1.15)	−0.78 (1.08)	−0.81 (209), 0.42
Executive function composite	−0.66 (0.89)	−0.60 (0.90)	−0.47 (209), 0.64

Except for education and gender, values are listed as mean (SD).

*MCI* mild cognitive impairment, *MCI+rMDD* mild cognitive impairment + remitted major depressive disorder, *MMSE* Mini-Mental State Examination, *MoCA* Montreal Cognitive Assessment, *TGC* Theta-gamma coupling, *t* independent samples *t* test, *χ*^2^ Chi square test, *df* degrees of freedom.

There were no differences in the demographic variables between the MCI and MCI+rMDD groups (*p*s > 0.05). The MoCA scores were statistically higher in the MCI+rMDD group (mean: 24.70, SD: 2.61) than in the MCI group (mean: 23.80, SD: 2.43; *t*(208) = −2.56, *p* = 0.01).

### Determining the TGC cutoff

The TGC cutoff that best separated our whole sample into 2-back impaired and not impaired performers was 0.0021 [0.0012, 0.0024], with a sensitivity of 82% [45%, 90%] and a specificity of 42% [33%, 80%]. The Youden Index at this cutoff was 0.24 [0.14, 0.38].

### Cognitive performance in the TGC and diagnostic groups

**Fig. 1:**
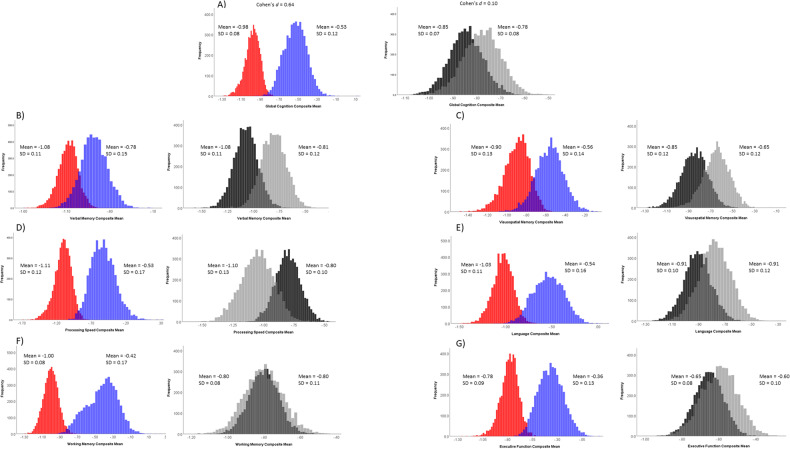
Histograms demonstrating the distributions of the average *z* score for each cognitive domain for the primary analyses across the 5000 bootstrapped iterations for the low (red) vs. high (blue) TGC groups and the MCI (dark gray) vs. MCI+rMDD (light gray) groups. **A** global cognition composite; **B** verbal memory composite; **C** visuospatial memory composite; **D** processing speed composite; **E** language composite; **F** working memory composite; **G** executive function composite.

The results for the primary analysis are presented in Table 3 and Fig. 1. As hypothesized, our primary analysis revealed that for global cognition, the Cohen’s *d* for the difference between the two TGC groups (Cohen’s *d*_TGC_ = 0.64, [0.32, 0.88]) was larger than the Cohen’s *d* for the difference between the diagnostic groups (Cohen’s *d*_diagnosis_ = 0.10 [0.004, 0.37]; Cohen’s *d*_difference_ = 0.54, [0.10, 0.80]). We also found that the difference between the TGC groups (Cohen’s *d*_TGC_ = 0.73, [0.24, 0.96]) was larger than the difference between the diagnostic groups (Cohen’s *d*_diagnosis_ = 0.001 [0.005, 0.32]) for the working memory domain (Cohen’s *d*_difference_ = 0.73, [0.09, 0.88]).

**Table 3:** Cohen’s *d* for differences between the TGC groups and the diagnostic groups.

	Cross-validation analysis in bootstrapped samples					
	Cohen’s *d* for TGC groups^a^ [95% CI]	Cohen’s *d* for diagnostic groups [95% CI]	Difference between Cohen’s *d* for TGC and diagnostic groups [95% CI]	Cohen’s *d* for TGC groups^b^ [95% CI]	Cohen’s *d* for diagnostic groups [95% CI]	Difference between Cohen’s *d* for TGC and diagnostic groups [95% CI]
Global cognition composite	0.64 [0.32, 0.88]	0.10 [0.004, 0.37]	0.54 [0.10, 0.80]	0.57 [0.15, 0.95]	0.17 [0.01, 0.48]	0.39 [−0.14, 0.85]
Verbal memory	0.22 [0.02, 0.55]	0.22 [0.16, 0.47]	0 [−0.35, 0.42]	0.27 [0.01, 0.65]	0.25 [0.01, 0.59]	0.02 [−0.47, 0.52]
Visuospatial memory	0.22 [0.02, 0.56]	0.16 [0.01, 0.42]	0.09 [−0.27, 0.46]	0.28 [0.02, 0.64]	0.21 [0.01, 0.51]	0.08 [−0.34, 0.52]
Processing speed	0.54 [0.20, 0.81]	0.20 [0.02, 0.47]	0.29 [−0.07, 0.65]	0.49 [0.07, 0.92]	0.24 [0.1, 0.59]	0.25 [−0.25, 0.75]
Language	0.55 [0.12, 0.75]	0.12 [0.006, 0.39]	0.43 [−0.12, 0.67]	0.43 [0.04, 0.82]	0.19 [0.01, 0.50]	0.24 [−0.28, 0.71]
Executive function	0.49 [0.15, 0.75]	0.06 [0.005, 0.34]	0.43 [−0.02, 0.67]	0.43 [0.05, 0.82]	0.17 [0.01, 0.46]	0.26 [−0.21, 0.73]
Working memory	0.73 [0.24, 0.96]	0.001 [0.005, 0.32]	0.73 [0.09, 0.88]	0.57 [0.07,1.02]	0.16 [0.01, 0.45]	0.41 [−0.14, 0.90]

^a^Based on a TGC cutoff of 0.0021 [0.0012, 0.0024] with a sensitivity of 82% [45%, 90%], a specificity of 42% [33%, 80%], and a Youden index of 0.24 [0.14, 0.38].

^b^Based on a TGC cutoff of 0.0019 [0.0012, 0.0026], with a sensitivity of 71% [38%, 93%], a specificity of 49% [22%, 81%], and a Youden index of 0.2 [0.02, 0.39].

## Results from the cross-validation analysis

**Fig. 2:**
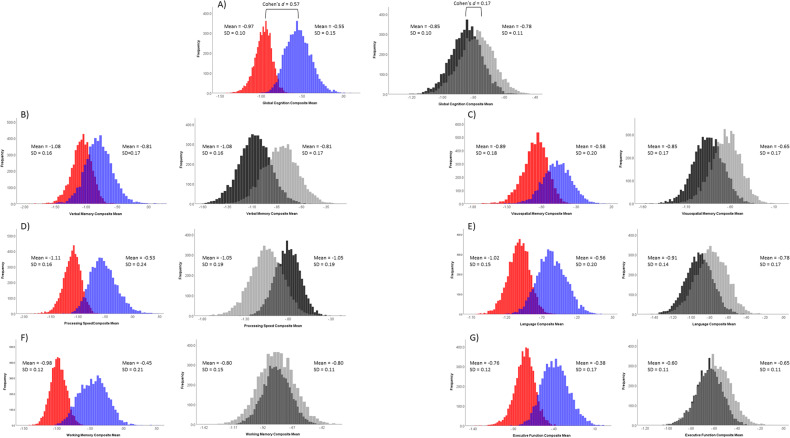
Histograms demonstrating the distributions of the average z score for each cognitive domain for the cross-validation analyses across the 5000 bootstrapped iterations for the low (red) vs. high (blue) TGC groups and the MCI (dark gray) vs. MCI+rMDD (light gray) groups. **A** global cognition composite; **B** verbal memory composite; **C** visuospatial memory composite; **D** processing speed composite; **E** language composite; **F** working memory composite; **G** executive function composite.

The results from the cross-validation analysis are also presented in Table 3 and Fig. 2. While there were no significant differences between the two types of groups, the results were comparable to our primary analyses results in magnitude and direction.

## Discussion

The aim of this study was to determine whether prefrontal cortex TGC better differentiates individuals with MCI, with or without rMDD, on global cognition than their clinical diagnosis. Our results support our hypothesis with two main findings: (1) there was little difference between the two diagnostic groups MCI and MCI+rMDD with regard to global cognition or any cognitive domains; (2) using a TGC cutoff, there were large differences between the high-TGC vs. low TGC- with regard to global cognition and working memory.

Cognitive performances did not differ between participants with MCI and those with MCI+rMDD. Past research examining differences in these two diagnostic groups is sparse, and only a few studies have directly compared the cognitive function of these two groups. Our results are congruent with one study that showed no difference in MMSE scores between those with MCI and those with MCI+rMDD [5]. Another study did find differences between these two groups in global cognition, processing speed, and executive function [4]; however, this study included participants with rMDD and MDD in an acute MDE. It is possible this heterogeneous group has more cognitive impairment than a group with rMDD alone. This is consistent with the literature on MCI with or without acute depressive symptoms. Individuals with MCI and depressive symptoms have been shown to be more impaired than those with MCI without depressive symptoms in several cognitive domains, including executive functioning [7, 34], memory [35, 36], and attention [7, 36]. Several studies looking at biological markers closely associated with cognition have also found mixed results when comparing those with MCI and MCI+rMDD. In an overlapping sample of participants with the current study, those with MCI+rMDD have been shown to have higher scores on an index of accelerated aging compared to those with MCI only [37]. In contrast, two MRI studies using overlapping samples with our study demonstrate no difference between resting state functional connectivity in the executive-control network in those with MCI compared to those with MCI+rMDD [38], whereas individuals with MCI+rMDD had better mean diffusivity in a frontal-executive white matter tract than those with MCI alone [39]. Taken together, our findings and the literature suggest that cognitive performance does not differ between individuals with MCI and those with MCI+rMDD. This suggests that clinical diagnosis might provide little information with respect to cognitive functioning and possible risk for future cognitive decline. Thus, clinical diagnoses may not be the right approach when it comes to examining cognitive function and, the possible risk for cognitive decline. This underscores the need for a biomarker-based cognitive classification instead of one based solely on clinical diagnosis.

In contrast to the diagnosis-based separation, we did observe differences in global cognition using a sample-derived TGC cutoff, indicated by a moderate to large Cohen’s *d* value. Exploratory analyses also show moderate to large Cohen’s *d* for working memory. These findings support our hypotheses that prefrontal TGC is indexing prefrontal cortical function, as the frontal lobes are critical in working memory and overall executive functioning [40–45]. Executive dysfunction is common in MCI [46], and can be predictive of those more likely to experience cognitive decline or develop dementia [47, 48]. In a study of 482 patients with amnestic MCI, patients with frontal-executive dysfunction, had a higher risk of progression to dementia than those with language or visuospatial dysfunction. In addition, those with frontal-executive dysfunction showed greater cortical thinning, particularly in the frontal region [49]. In a recent neuroimaging study, their MCI sample was split between those with low vs. high executive functioning [50]. Compared to control participants, the high executive functioning group demonstrated impaired regional brain activity, but intact functional connectivity in the executive-control network. By contrast, in the low executive functioning group, both regional activity and functional connectivity were impaired. Further, there was a negative association between impaired executive functioning and both regional brain activity and functional connectivity. The authors concluded that the functional integrity of the executive-control network may contribute to the retention of executive function in MCI. These two studies provide evidence that individuals with MCI with executive dysfunction have altered the structure and function of the frontal cortex. Thus, if prefrontal cortex TGC is an index of executive functioning, then those with lower TGC and executive functioning could be at higher risk for future cognitive decline or progression to dementia than those with higher TGC, possibly due to cortical thinning or functional disconnection in the frontal cortex due to neurodegenerative disease or other mechanisms.

We note four limitations to our study. First, we recognize the limitation of our primary approach of generating and testing our TGC cutoff in the same sample of participants. To mitigate this limitation, we conducted a cross-validation analysis using bootstrapping and splitting our sample into training and validation samples. The trend from this analysis was similar to that of our primary analyses, and showed that TGC could separate individuals with MCI and MCI+rMDD on cognition better than their clinical diagnosis. However, the differences in Cohen’s *d* values between these approaches were not significant in our cross-validation analysis. This is possibly related to a relatively small sample size when we split the sample in half. Second, rMDD was established based on a distant history of a major depressive episode and not current symptoms. To mitigate this limitation, we required that either the depressive episode be within the past 5 years, or that there was evidence of medical care for the episode, e.g., hospitalization. Third, the sensitivity, specificity, and Youden Index values for our TGC cutoff differentiating individuals into “impaired” vs. “not impaired” on 2-back performance were lower than we would have liked. Ideal sensitivity/specificity values would have been 80% or higher, with a Youden Index ≥0.6. Still, the main goal in this study was not to characterize the TGC cutoff in separating individuals on the 2-back task, but in separating groups defined by the TGC on other cognitive functions. Last, our study is cross-sectional and, therefore, we cannot make conclusions with respect to cognitive decline but only with respect to cognitive impairment as a possible proxy for cognitive decline. Follow-up longitudinal analyses are needed.

In conclusion, our study suggests that prefrontal TGC could be a promising marker for identifying individuals at higher risk for cognitive decline. Future longitudinal studies are needed to confirm the utility of this neurophysiologic marker.

## Data Availability

The data used in the current publication is available upon request.
